# Later sleep timing predicts accelerated summer weight gain among elementary school children: a prospective observational study

**DOI:** 10.1186/s12966-021-01165-0

**Published:** 2021-07-12

**Authors:** Jennette P. Moreno, Javad Razjouyan, Houston Lester, Hafza Dadabhoy, Mona Amirmazaheri, Layton Reesor-Oyer, Teresia M. O’Connor, Daphne C. Hernandez, Bijan Najafi, Candice A. Alfano, Stephanie J. Crowley, Debbe Thompson, Tom Baranowski

**Affiliations:** 1 Department of Pediatrics, USDA/ARS Children’s Nutrition Research Center, Baylor College of Medicine, 1100 Bates Street, Houston, TX 77030 USA; 2 VA HSR&D Center for Innovations in Quality, Effectiveness and Safety, Michael E. DeBakey VA Medical Center, Houston, TX 77030 USA; 3 Department of Medicine, Institute for Clinical and Translational Research, Baylor College of Medicine, Houston, TX 77030 USA; 4 Big Data Scientist Training Enhancement Program (BD-STEP), VA Office of Research and Development, Washington, DC USA; 5 Department of Management, University of Mississippi, Oxford, MS USA; 6 Department of Surgery, Interdisciplinary Consortium on Advanced Motion Performance (iCAMP), Baylor College of Medicine, Houston, TX USA; 7 Department of Exercise Science, Arnold School of Public Health, University of South Carolina, Columbia, SC USA; 8 Cizik School of Nursing, University of Texas Health Science Center at Houston, Houston, TX USA; 9 Department of Psychology, Sleep and Anxiety Center of Houston (SACH), University of Houston, Houston, TX USA; 10 Department of Psychiatry & Behavioral Sciences, Biological Rhythms Research Laboratory, Rush University Medical Center, Chicago, IL USA

## Abstract

**Objectives and background:**

Social demands of the school-year and summer environment may affect children’s sleep patterns and circadian rhythms during these periods. The current study examined differences in children’s sleep and circadian-related behaviors during the school-year and summer and explored the association between sleep and circadian parameters and change in body mass index (BMI) during these time periods.

**Methods:**

This was a prospective observational study with 119 children ages 5 to 8 years with three sequential BMI assessments: early school-year (fall), late school-year (spring), and beginning of the following school-year in Houston, Texas, USA. Sleep midpoint, sleep duration, variability of sleep midpoint, physical activity, and light exposure were estimated using wrist-worn accelerometry during the school-year (fall) and summer. To examine the effect of sleep parameters, physical activity level, and light exposure on change in BMI, growth curve modeling was conducted controlling for age, race, sex, and chronotype.

**Results:**

Children’s sleep midpoint shifted later by an average of 1.5 h during summer compared to the school-year. After controlling for covariates, later sleep midpoints predicted larger increases in BMI during summer, (γ = .0004, *p* = .03), but not during the school-year. Sleep duration, sleep midpoint variability, physical activity levels, and sedentary behavior were not associated with change in BMI during the school-year or summer. Females tended to increase their BMI at a faster rate during summer compared to males, γ = .06, *p* = .049. Greater amounts of outdoor light exposure (γ = −.01, *p* = .02) predicted smaller increases in school-year BMI.

**Conclusions:**

Obesity prevention interventions may need to target different behaviors depending on whether children are in or out of school. Promotion of outdoor time during the school-year and earlier sleep times during the summer may be effective obesity prevention strategies during these respective times.

**Supplementary Information:**

The online version contains supplementary material available at 10.1186/s12966-021-01165-0.

## Introduction

Increases in standardized body mass index (BMI) have been demonstrated among elementary school age children during summer months across the United States [1–7] and Japan [8–10]. Two large scale studies have demonstrated that accelerated increases in BMI during summer contributed to increased rates of overweight and obesity among elementary school students [7, 11]. However, most obesity prevention interventions take place during the school year, ignoring summer [12]. Further, most interventions targeting traditional energy balance related behavior have been largely ineffective [13], suggesting a need to more closely consider the contexts during which accelerated weight gain occurs and the behaviors associated with weight gain across various contexts. (i.e., school year and summer environments).

Examinations of the causes of accelerated summer weight gain have primarily explored differences in children’s energy balance related behaviors during the school year and summer [14] and few have investigated how differences in behaviors during the school year and summer relate to children’s change in BMI during those periods. In a secondary analysis of a cluster randomized controlled trial conducted among urban, public elementary schools in Massachusetts, researchers examined the association between dietary intake and physical activity (PA) during the school year and summer and changes in BMI during those times [15, 16]. Overall, children gained BMI at a faster rate during summer compared to the school year [15]. During summer, children ate fewer servings of fruits and vegetables and more servings of salty snacks, sweets, and sugar sweetened beverages; however, diet did not mediate the relationship between season and mean monthly BMI change [15]. Likewise, during summer, children achieved eight fewer minutes of moderate to vigorous PA and increased their sedentary behavior by 28 min. However, neither PA nor sedentary behavior mediated the relationship between season and BMI change [15]. Given the small sample size, there is a need to replicate these findings in a larger sample. However, these findings also suggest a need to consider the potential of alternative behaviors or factors, such as sleep, to contribute to unhealthy weight gain during summer.

There is increasing awareness of the importance of adequate and consistent sleep that occurs at a time when the body is expecting it for maintaining a healthy BMI [17–21]. Comparisons of children’s sleep timing and sleep duration across seasons suggests that children exhibit later sleep timing during summer compared to other seasons [22–24]. In fact, later parent reported sleep timing has been shown to moderate the relationship between parent reported sleep duration and concurrent BMI in preschool-age children [25]. However, the mechanisms through which sleep moderates weight gain are not well understood.

The Circadian and Circannual Rhythms Model (CCR Model) of accelerated summer weight gain, proposed that in the absence of the social demands of the school year (e.g., school schedules and sleep related parenting practices), children would exhibit changes in behavior such as shortened sleep duration, later and more variable sleep timing, and reduced PA [26, 27]. It was hypothesized that reductions in PA reduce the amplitude of the circadian system and sleep/wake homeostasis, thereby reducing the pressure to sleep at night [28, 29]. Lower levels of PA may also affect circadian rhythmicity through decreased exposure to natural light, as increased PA is associated with greater outdoor light exposure [30–32]. These changes in behaviors may affect synchronization of circadian and circannual rhythms by affecting children’s exposure to the light/dark cycle and contributing to a blunting of circadian rhythmicity which may contribute to accelerated weight gain [26, 27, 33]. According to the CCR Model children’s sleep timing, duration, and variability, PA, and light exposure represent novel pathways which may differentially influence children’s BMI during the school year and summer. However, the extent to which seasonal or school-summer differences in objectively measured sleep and light exposure patterns are related to changes in children’s weight status during the school year and summer holiday is unknown. This study addresses this gap and provides a preliminary test of the CCR Model.

The current study examined differences in children’s sleep duration, sleep timing, variability of sleep timing, PA, and exposure to outdoor light during the school-year and summer holiday. In addition, this study explored these sleep and circadian related parameters as predictors of school-year and summer change in BMI among 5–8 year-old children. It was hypothesized that children would demonstrate shorter sleep duration, later and more variable sleep timing, and lower levels of PA during the summer holiday compared to the school-year, which would, in turn, be associated with accelerated summer weight gain.

## Methods

### Study design and setting

A prospective, observational cohort study was conducted in Houston, TX, USA, a large subtropical urban area (29.8° N, 95.4° W), to examine differences in the rates of BMI change during the school-year and summer holiday (typically June through mid-August), as well as differences in children’s sleep, light exposure, and PA. BMI data were collected at three time points: early in the school-year, late in the school-year, and late summer, (i.e., the study period spanned September, 2016–October, 2017). Behavioral assessments were conducted during the school-year and summer holiday (see Table 1). All children attended local elementary schools (i.e., primary school) for the 2016–2017 school-year, which extended from about 8/22/2016–6/1/2017 and the summer holiday that lasted until 8/22/2017. Hurricane Harvey affected the Houston area from 8/25/2017–8/29/2017. School closures lasted approximately from 8/24/2017–9/11/2017. None of the participants reported experiencing significant flooding or loss due to the storm. The Institutional Review Board at Baylor College of Medicine approved the study protocol (H-39431).

**Table 1:** Measurement Timeline

Measures	Baseline Fall semester 2016	End of School-Year 2017	Summer Holiday 2017	Beginning of School-Year 2017
**Body Composition**				
Height	✓	✓		✓
Weight	✓	✓		✓
BMI (calculated)	✓	✓		✓
**Accelerometry**				
Sleep	✓		✓	
Physical Activity	✓		✓	
Ambient Light Exposure	✓		✓	
**Other**				
Sleep diary and accelerometer wear log	✓		✓	
Chronotype			✓	

### Study participants

Because early elementary school (kindergarten-2nd grade or 5–8 year-olds) was a period when accelerated summer weight gain emerged in previous studies [11], eligibility criteria for parent-child dyads included being a parent or caregiver of a 5–8 year-old child in kindergarten through 2nd grade. The child had to be enrolled in a school with a 10–12 week summer holiday and have the ability to participate in physical education. Exclusion criteria included the child having a medical condition affecting diet, PA, sleep, or weight (e.g., celiac disease, diabetes, ADHD, sleep apnea, sleep disorders), being a homeschooled student, attending a year round school, having been held back 2 or more grade levels, and planning to move from the Houston area.

### Recruitment

Participants were recruited through flyers distributed at elementary schools and from a volunteer database. Parents were told that this was a study about children’s sleep and weight gain during the school-year and summer and that compensation for participation would be provided. Parents were directed to a website for additional information. Interested parents provided consent to proceed with the online eligibility screener. Eligible families were scheduled for an appointment in which the study was described in detail and written informed consent and assent were obtained.

### Procedures

Anthropometric data (height and weight) were obtained by trained research staff during the fall semester (range: 9/07/16–12/03/2016, mean: 10/19/2016), later part of the spring semester (range: 04/01/2017–6/07/2017, mean: 4/20/2017), and 3) beginning of following school-year (range: 08/18/2017–10/16/2017, mean: 9/11/2017). Children’s heights were measured without footwear using a Holtain stadiometer. Weights were assessed in light clothing without footwear using a Healthometer digital scale. Because BMI is the preferred proxy measure for change in fat mass over intervals of less than 1 year, BMI (kg/m^2^) was computed [34–36].

At baseline, Actigraphs (GT3X-BT, Pensacola, FL) were used to assess children’s sleep and PA during the school-year. Dates of the school-year accelerometer assessment ranged from 9/16/16–12/12/16 and an additional 9 children completed their accelerometer assessment in May 2017. Data were not collected during weeks when there was a school holiday. Additionally, accelerometry was not collected the week of or after Daylight Saving Time. Actigraphs were mailed to families during the summer holiday. Parents also completed a sleep diary and accelerometer wear log [37–39]. Accelerometers were mailed to families during a week when children were not in summer school or out-of-town on a trip. Dates of the summer accelerometer assessment ranged from 6/2/17–8/16/17. A link to an online instructional video demonstrated proper wear and how to avoid covering the accelerometer with clothing [40]. Families were compensated up to $145 for completion of all assessments and travel.

Previous research has shown that at least 5 nights of actigraphy are needed to provide a reliable estimate of children’s sleep duration and timing, and that a week of data is needed to ensure 5 nights of usable data due to factors such as wear time, technical issues, or co-sleeping [37]. As a result, at each assessment, children wore the Actigraph GT3X-BT monitor for 7 days and 8 nights with the goal of attaining at least 5 nights of scorable sleep data [37]. In accordance with the Society of Behavioral Sleep Medicine guidelines, monitors were worn on the wrist of their non-dominant hand [41, 42], attached using a wristband that allowed adequate monitor-to-skin contact. The Actigraph GT3X-BT is a tri-axial microelectromechanical systems accelerometer. The monitor digitized acceleration data using a 12-bit analog to a digital converter with a sampling rate of 30 Hz. Data were downloaded using Actigraph’s digital pass filter with a band width of .25 Hz–2.5 Hz, designed to detect normal human behavior. Wear time data were also collected by the GT3X-BT monitor.

The Sadeh algorithm was used to score epochs as sleep or wake [43–45]. According to established protocols, each sleep episode reported in the parent diary was inspected in the activity data starting 15 min before and 15 min after the reported bedtime and wake time, respectively [37, 38, 46]. If epochs of low activity existed outside of the scoring interval or if non-wear time occurred during the interval, a consensus was reached by the research team. Nights were considered valid if the participant provided 20 min of wear time before sleep onset. Non-wear time in the hour before bedtime had to be less than 60 min unless confirmed by the wear log, or unless ambient light data were available to confirm bedtime. Sleep midpoint was defined as the midpoint between sleep onset and offset (see Table 2). Sleep midpoint was selected as a measure of sleep timing as it takes into account both bedtime and waketime and is also strongly correlated with endogenous measures of circadian timing such as dim light melatonin onset and core body temperature [47]. Total sleep time was defined as the total number of minutes scored as sleep between sleep onset and offset. The intraindividual standard deviation of sleep midpoint across nights represented variability of the timing of children’s sleep patterns [48].

**Table 2:** Definition of Sleep Parameters

Sleep Parameter	Definition
Sleep onset	The first minute of the first three consecutive epochs scored as sleep
Sleep offset	The last minute of at least 5 consecutive minutes of sleep occurring before 15 min after the reported wake-up
Sleep Midpoint	Midpoint between sleep onset and sleep offset
Total sleep time	total number of minutes scored as sleep between sleep onset and offset
Intraindividual Variability of Sleep Midpoint	Root Mean Square of Successive Differences of Sleep Midpoint The square root of the average difference in sleep midpoint across successive nights^a^

^a^$$ \sqrt{\left[\left(\mathrm{night}\ 2-\mathrm{night}\ 1\right)+\left(\mathrm{night}\ 3-\mathrm{night}\ 2\right)+\dots +\left(\mathrm{night}\ \mathrm{n}-\mathrm{night}\ \mathrm{n}-1\right)\right]/\mathrm{n}} $$

After removing the sleep periods and non-wear time [49], activity counts captured in 60-s epochs were categorized into sedentary, light, and moderate to vigorous PA using established cut points [50, 51]. In order to consider the impact of differences in wear time, percent time spent in sedentary, light, and moderate to vigorous activity was calculated based on total wear time, excluding sleep periods. Valid days were defined as at least 10 h of wear time in a 24-h period [52]. The photocell contained in the Actigraph GT3X-BT is capable of measuring 0–5000 lx and measured ambient light exposure. Lux data were binned in 60-s epochs. A cut point of 240 lx signified exposure to outdoor light [53]. Percentage of time spent outside was calculated as the number of epochs of light exposure greater than or equal to 240 lx divided by total number of epochs between sleep offset (wake time) and sleep onset (bedtime) multiplied by 100. To be included in analyses, participants had to provide at least 5 valid nights and 4 valid days of accelerometer data [37, 52]. At least one of the valid days and nights had to be a weekend day (Saturday or Sunday) or weekend night (Friday or Saturday) and at least two of the valid days and nights had to be a weekday (Monday–Friday) or weeknight (Sunday–Thursday).

Chronotype (i.e., the behavioral expression of the biological clock or the tendency to sleep at a certain time) was assessed using the 5-point chronotype score from the Children’s Chronotype Questionnaire [54]. Parents provided parent and child demographic characteristics.

### Sample size

Power calculations were based on detecting a small but clinically meaningful difference in children’s sleep-wake patterns between the school-year and summer. While no data regarding seasonal differences in sleep timing were available, a previous study demonstrated that children slept an additional 40.5 min in the winter compared to summer [24]. Power calculations assumed nominal values for the Type I and II error rates (i.e., 5 and 20%, respectively; two tailed). Sample size and power estimates were calculated using G*Power 3.1 (Franz Faul, Universität Kiel, Germany). Based on these assumptions, 108 participants were needed to detect within-subjects differences in sleep between the school-year and summer.

### Statistical design and analysis

**Fig. 1:**
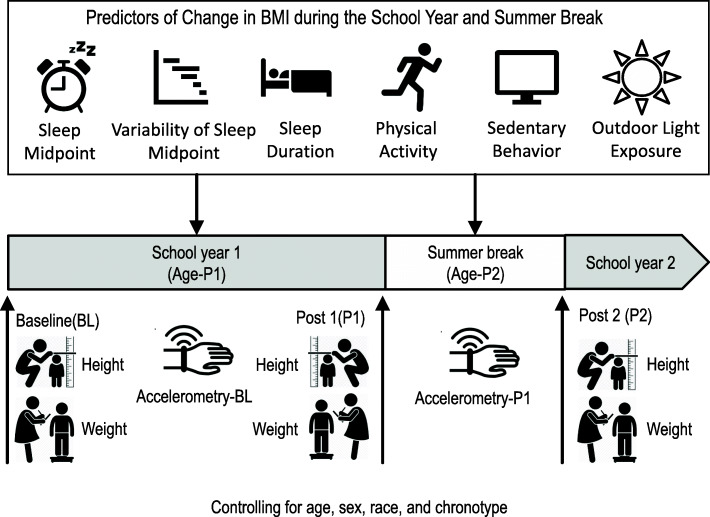
Conceptual Model

Descriptive analyses were conducted using mixed-effects analysis of variance to test for differences between males and females across repeated measures of anthropometric, sleep, PA, and light exposure parameters using SPSS software version 27(©2020, IBM Corp., Armonk, NY). Mixed models were performed to compare differences in children’s sleep midpoint and duration; variability of sleep midpoint; hours of outdoor light exposure, sedentary behavior, light and moderate to vigorous PA; and change in BMI during the school-year and summer using SAS software version 9.4 (©2013, SAS Institute Inc., Cary, NC). The significance level was set at *p* < .006 to control for multiple testing in these 8 comparisons. To examine the effect of sleep duration, sleep midpoint, sleep variability, PA level, and percentage of wake time exposed to outdoor light on change in BMI, growth curve modeling was used to estimate inter-individual variability in intra-individual patterns of change over time. Specifically, individual differences in intercepts and slopes for BMI during the school-year (i.e., change in age at end of the school-Year 2017 (Age-P1)) and the summer (i.e., change in age beginning of the school-year 2017 (Age-P2)) were conducted controlling for age, sex, race, chronotype (Fig. 1). The effect of age was allowed to vary across participants and controlled for differences in the number of days between BMI assessments across participants. Variables were selected a priori for inclusion in the model based on theory [26, 27] and previous research demonstrating differences in children’s sleep across sex [55], race [56], age [57], and chronotype [58]. Using a theoretically driven step-down modeling approach, variables were tested to determine if they should be included as a covariate or allowed to interact with the school-year (Age-P1) and summer slopes (Age-P2). If the interaction terms (Age-PX x Predictor) did not improve the fit of the model (i.e., increase in the variance explained), the variable was included as a covariate (i.e., the variable was included with only a main effect). A significant interaction with Age-P1 indicated that the predictor variable was significantly associated with change in BMI during the school year and a significant interaction with Age-P2 indicated a significant association with change in BMI during the summer. In order to examine the impact of wear time on our findings, we also ran our models using percent time spent in sedentary behavior and light and moderate to vigorous PA. Due to multicollinearity of these three variables, percent time spent in light PA was excluded from the model examining these behaviors as predictors of change in BMI during the school year and summer. Significance level was set at *p* < .05. The analytic plan allowed for missing data in the outcome variables; however, participants were excluded if they had missing data on a predictor.

## Results

### Participants

A total of 256 participants were screened for eligibility. Reasons for exclusion are provided in eFigure 1. A total of 119 kindergarten through second graders met criteria for inclusion in the study and provided BMI data at baseline. The analytic sample was comprised of 95 children. Demographics of the overall sample and the analytic sample are presented in Table 3. Participants were not excluded from analyses because of missing BMI data; however, 119, 114 and 110 participants provided BMI data at baseline, the end of the school year and beginning of the following school year. There was an average of 183 ± 28 days between BMI assessments during the school year and 143 ± 18 days during the summer.

**Table 3:** Baseline Demographic Characteristics (*Mean* (*SD*) or %(*n*))

Variables	Overall Sample (***n*** = 119)	Analytic Sample^**a**^ (***n*** = 95)
**Child Age at Baseline**	7.0(0.88)	6.9(0.82)
**Child Sex (% female)**	48 (57)	46 (44)
**Race/Ethnicity**		
Latino	24(29)	23(22)
Non-Hispanic Black	24 (28)	24 (23)
Non-Hispanic White	19 (23)	22(21)
Asian/Asian American	19 (23)	19(18)
Multiracial	8 (10)	8 (7)
Other	3 (3)	3 (3)
Hispanic Black	3 (3)	1 (1)
**Household Income**		
Less than $70,000	41 (49)	42 (40)
$70,000 or more	48 (57)	46 (44)
Declined to provide	11 (13)	12 (11)
**Parent Education**		
Some College or less	31 (37)	28 (27)
College Degree	43 (51)	45 (43)
Graduate School	26 (31)	26 (25)
**Weight Classification**		
Healthy Weight	70 (83)	69 (66)
Overweight	15 (18)	17 (16)
Obese	15 (18)	14 (13)

^a^There were no significant differences between the overall sample and the analytic sample

### Descriptive school-summer differences in behavioral rhythms and change in BMI

BMI increased significantly over time, *F* (2, 94) = 17.04, *p* < .001, with significant gains during both the school-year, *t* (94) = 4.21, *p* < .001, and summer, *t* (94) = 4.81, *p* < .001 (eTable 1). There was no difference in change in BMI during the school-year and summer, *t* (94) = .10, *p* = .273 (Table 4). During summer, the average sleep midpoint shifted 87 min later than during the school-year, *t* (94) = − 12.18, *p* < .001. On average, children went to bed around 9:42 PM ± 47 min and woke up at 6:52 AM during the school-year. Total sleep time decreased by 11.78 min in summer, *t* (94) = 3.23, *p* = .002. Children demonstrated a similar amount of variability in sleep midpoint during the school-year and summer, *t* (94) = − 0.55, *p* = .581. Additionally, when adjusting for multiple comparisons, children were exposed to similar amounts of outdoor light during the school-year compared to summer, *t* (94) = − 2.03, *p* = .045. During summer, children increased their sedentary behavior by 32 min, *t* (94) = − 3.25, *p* = .002, and decreased their light PA by 57 min, *t* (94) = 7.46, *p* < .001. There were no differences in children’s moderate to vigorous PA during the school year and summer *t* (94) = − 2.65, *p* = .010, when adjusting for multiple comparisons. The direction and significance level of the results did not differ when examining differences in the percentage of time spent in sedentary behavior and light and moderate to vigorous PA during the school year and summer (results not shown). Additional descriptive information regarding children’s sleep schedules and PA levels during the school-year and summer are provided in eTable 2.

**Table 4:** School Summer Differences in Growth, Sleep, and Energy Balance Related Outcomes

Dependent Variable	School-Year ***Mean*** (***SD***)	Summer Holiday ***Mean*** (***SD***)	Significance Level (***p*** value)^**a**^
BMI Change	0.28 (0.66)	0.39 (0.77)	0.27
**Sleep Midpoint (hrs.)**	**2:17 AM (0.65)**	**3:44 AM (1.44)**	**< 0.001**
**Total Sleep Time (hrs.)**	**7.86 (0.50)**	**7.66 (0.67)**	**0.002**
Variability Sleep Midpoint^b^ (min.)	51.0 (30.5)	53.5 (40.6)	0.580
Exposure to Outdoor Light^c^ (hrs.)	1.02 (0.49)	0.89(0.52)	0.045
**Sedentary Behavior (min.)**	**446.5 (69.3)**	**478.6 (100.0)**	**0.002**
**Light PA (min.)**	**361.9 (49.6)**	**304.7 (78.4)**	**< 0.001**
Moderate to Vigorous PA (min.)	67.86 (26.1)	60.06 (30.5)	0.010

^a^Significance level was set at *p* < .006 to control for multiple testing across these 8 variables of interest

^b^Root Mean Square of Successive Differences of Sleep Midpoint

^c^Outdoor light exposure defined as 60 s epochs with ambient light exposure > 240 lx

### Predictors of BMI change

The modeling process revealed the following findings for weight gain in the summer: later sleep midpoints resulted in larger BMI gains, (γ = .0004, *t* (88.4) = 2.16, *p* = .034), and females tended to increase their BMI at a faster rate compared to males, γ = .06, *t* (87.2) = 2.00, *p* = .049 (see Table 5). In the school-year, increased outdoor light exposure was associated with less weight gain, γ = −.01, *t* (91.9) = − 2.47, *p* = .015. Results did not differ when including percentage of time spent in sedentary behavior and moderate to vigorous activity during the school year and summer in the model (see eTable 3). Overall, this model explained 27.72% of BMI variation across the study period.

**Table 5:** Predictors of Change in BMI during the School-Year and Summer

Model Parameter	*Estimate*	*SE*	*DF*	*t*	*p*^a^	*95% CI*	
						*LL*	*UL*
Intercept	11.50	3.62	71.10	3.18	0.00	4.28	18.71
^b^Chronotype: Definitely a Morning Type	0.82	1.45	70.80	0.57	0.57	−2.07	3.71
Chronotype: Rather a Morning Type than an Evening Type	1.48	1.44	70.80	1.03	0.31	−1.38	4.35
Chronotype: Neither a Morning nor an Evening Type	1.16	1.52	70.80	0.76	0.45	−1.87	4.19
Chronotype: Rather an Evening Type than a Morning Type	0.89	1.44	70.90	0.62	0.54	−1.98	3.76
Chronotype: Definitely an Evening Type	1.87	1.92	71.00	0.97	0.33	−1.96	5.70
^c^Race: African American	−0.95	1.04	71.50	−0.91	0.36	−3.02	1.12
Race: Caucasian	−0.48	0.94	71.70	−0.51	0.61	−2.34	1.39
Race: Asian	−1.53	1.06	71.60	−1.44	0.15	− 3.65	0.59
Race: Other	1.12	1.63	71.10	0.69	0.49	−2.13	4.37
Sex	−0.19	0.61	72.60	− 0.32	0.75	−1.41	1.03
Age-BL^d^	0.37	0.33	71.00	1.12	0.27	−0.29	1.02
**Age-P1**^**e**^	**0.04**	**0.01**	**92.70**	**4.13**	**<.0001**	**0.02**	**0.07**
**Age-P2**^**f**^	**0.05**	**0.02**	**88.50**	**2.40**	**0.02**	**0.01**	**0.10**
**Sex x Age-P2**	**0.06**	**0.03**	**87.20**	**2.00**	**0.05**	**0.00**	**0.13**
Total Sleep Time-School	0.00	0.01	71.30	0.03	0.97	−0.02	0.02
Total Sleep Time-Summer	0.01	0.01	71.10	1.40	0.17	0.00	0.03
Variability of Sleep Midpoint (RMSSD^g^)-Summer	0.00	0.01	71.00	−0.01	0.99	−0.02	0.02
Sleep Midpoint-Summer	0.01	0.00	72.70	1.62	0.11	0.00	0.01
**Age-P2 x Sleep Midpoint-Summer**	**0.00**	**0.00**	**88.40**	**2.16**	**0.03**	**0.00**	**0.00**
Sedentary Behavior-School	0.01	0.01	71.00	1.11	0.27	−0.01	0.02
Light PA^h^-School	0.01	0.01	71.10	1.08	0.28	−0.01	0.03
Moderate to Vigorous PA-School	0.02	0.01	71.60	1.31	0.20	−0.01	0.04
Sedentary Behavior-Summer	0.00	0.00	71.10	−0.19	0.85	−0.01	0.01
Light PA-Summer	0.00	0.00	70.90	0.15	0.88	−0.01	0.01
Moderate to Vigorous PA-Summer	−0.02	0.01	70.90	−1.53	0.13	−0.04	0.01
Percentage of Time Outdoors-School	−0.08	0.14	73.30	−0.58	0.56	−0.36	0.20
Percentage of Time Outdoors-Summer	0.07	0.11	71.20	0.65	0.52	−0.15	0.30
**Age-P1 x Percentage of Time Outdoors-School**	**−0.01**	**0.01**	**91.90**	**−2.47**	**0.02**	**−0.02**	**0.00**

These are the results from a single growth curve model used to estimate individual differences in intercepts and slopes for BMI during the school-year and the summer. This model explained 27.72% of BMI variation across the study period (*R*^2^ = .27772)

^a^Significance level was set at *p* < .05. ^b^Reference category: I don’t know; ^c^Reference Category: Mixed Race; ^d^BL: Baseline Fall 2016; ^e^P1: End of the School-Year 2017; ^f^P2: Beginning of the School-Year 2017; ^g^RMSSD: Root Mean Square of Successive Differences; ^h^PA: Physical Activity

## Discussion

These findings confirmed that children shift their sleep timing later during summer compared to other seasons [22] and that later sleep timing contributed to shorter sleep durations among elementary school children who only partially compensated for later bedtimes with later wake times [24]. There was no difference in the variability of children’s sleep timing during the school year and summer. To our knowledge, this study was the first to explore the extent to which seasonal differences in sleep patterns were related to changes in children’s weight status during the school-year and summer. In the current study, having a later sleep midpoint during summer was associated with increases in BMI during summer; however, we did not observe a similar relationship between sleep timing and BMI during the school-year. Additionally, sleep duration and variability in sleep timing were not associated with change in BMI during the school-year or summer.

This study adds to a body of evidence suggesting that the later timing of sleep is associated prospectively with greater increases in BMI among children [59–61]. However, this association was observed only during summer. Consistent with the CCR Model, children’s sleep shifted over an hour and a half later during summer compared to the school-year. One possible explanation is that during out of school times like summer, children are permitted to stay up later, resulting in exposure to electrical light after sunset, thereby delaying the circadian clock [62], an effect suppressed during the school-year by parentally enforced bedtimes. Interestingly, adults demonstrated the opposite seasonal pattern, with earlier sleep onset, midpoint, and circadian timing in summer compared to winter [63–66]. The timing of sleep in adults was highly correlated with the timing of dawn [63, 66, 67] resulting in the shortening of sleep duration during summer [66].

Previous studies have found an association between children’s sleep duration and prospective change in BMI; however, the current study did not find an association between children’s school-year or summer sleep duration and prospective change in BMI during those times. Key differences between the current study and many previous studies include the use of accelerometry to estimate sleep [68] and follow-up periods of less than a year in the current study to assess prospective change in BMI [68, 69].

The CCR Model of accelerated summer weight gain proposed that the lack of social demands during summer would result in more variable sleep timing, which may increase the risk of circadian misalignment during summer contributing to accelerated summer weight gain [26, 27]. However, the current study found no evidence of school-summer differences in the variability of sleep timing and there was no association of variability in sleep timing with children’s change in BMI during the school year or summer. It is possible that we did not observe greater variability during summer because variability was examined across weekdays and weekends. It is likely that greater variability may have been observed if school-summer differences were compared across weekdays which are more heavily influenced by social demands, whereas weekends maybe more similar to summertime.

In a low-income, ethnically diverse sample of elementary school children, Tanskey et al. found that children decreased their MVPA by 8 min and increased their sedentary behavior by 28 min during summer using waist-worn accelerometers [15]. While our sample was also an ethnically diverse sample, most parents had attended college and reported an annual household income greater than $70,000. Similarly, in the current study using wrist-worn accelerometers, children decreased their moderate to vigorous PA by 8 min (not statistically significant), and their light PA by 57 min, while increasing their sedentary behavior by 32 min. While these results are consistent with previous findings, they are somewhat inconsistent with notions of summer as being a time when children have more time to play outside and be active. The Houston climate could have caused children to remain indoors to avoid the heat and humidity during summer, resulting in more sedentary behavior, less moderate to vigorous PA, and similar levels of light exposure compared to the school-year. However, most of the school-year accelerometer data were collected during the fall, when it is still quite warm, with the temperatures not becoming temperate until late October/early November. Additionally, similar results were observed in Massachusetts where the average summertime and fall temperatures are quite different than Houston [15], suggesting that factors other than weather are at play. It is also possible that during the school-year, compulsory activities (e.g., recess, commuting to and from school, and extracurricular school activities) may encourage greater outdoor time and vigorous PA and less sedentary time [70]. Nevertheless, the current study replicated Tanskey et al.’s findings that sedentary behavior and moderate to vigorous PA were unrelated to change in BMI during the school-year and summer in a larger sample [15]. Surprisingly, we found children were exposed to similar amounts of outdoor light during the summer and school-year. Greater time outdoors was associated with smaller increases in BMI, during the school-year. It is not clear why greater amounts of outdoor light during fall would be associated with smaller increases in BMI, though light exposure is known to affect energy expenditure and metabolism [71, 72].

In the current study, females increased their BMI at a faster rate during summer compared to males. There was no interaction between time and sex during the school year. While stratifying the analyses by sex was beyond the scope of the current analysis plan, exploring whether the association between children’s sleep and circadian rhythm-related behaviors and change in BMI vary by sex is an interesting avenue for future inquiry (see eTables 1 and 2).

The current study had several strengths including a within-subject longitudinal design, use of an objective measure of sleep, sedentary behavior and PA, and light exposure. Limitations included the inability to measure height and weight measurements on the first and last day of the school year and summer, making comparisons of change in BMI during the school year and summer less meaningful. To allow for comparison across these times periods, the number of days between assessments was controlled for in the analyses. Further, accelerometry provides an objective measure of sleep, but it lacks specificity to detect true wake and sleep, potentially resulting in the underestimation of sleep duration [43, 73–77]. To mitigate this limitation, parent reported bedtimes and wake times were used to identify sleep onset and offset [37, 38]. Because the measurement of sleep timing were the primary variables of interest in the current study, the actigraphs were worn on the wrist and activity counts were binned in 60 s epochs which is preferred for the measurement of sleep [41]. While empirically derived cut points for wrist-worn devices were used to estimate PA, the estimates of sedentary behavior and physical activity based on 60 s epochs may not have been optimal [42, 78]. Nevertheless, the school-summer differences in moderate to vigorous PA and sedentary behavior were remarkably like those observed when PA was assessed using the preferred 15 s epochs from waist worn devices [15, 78]. The ambient light sensor contained in the wrist worn Actigraph GT3x-BT may not accurately reflect light received by the photoreceptors of the eye. However, the Actigraph devices have been validated to discriminate between indoor and outdoor light exposure when worn by preschool age children [53]. Differences in children’s dietary intake and meal timing during the school-year and summer were not assessed or controlled for, limiting our ability to determine whether the later timing of sleep may have facilitated later mealtimes or additional evening snacks, resulting in accelerated weight gain [79]. While our sample was diverse in terms of race and ethnicity, our results may not generalize to lower income populations. Future research should attempt to replicate these findings across a broader range of socioeconomic levels. Additionally, the Houston area was affected by Hurricane Harvey during the collection of the final BMI measurement. While it is unclear what the impact of this may have been on children’s weight gain, it certainly resulted in a change in social demands, a need to remain indoors for about 4 days during the storm, and exposure to additional weeks of out of school conditions. Accelerometer data were not collected during the storm and thus the storm should not have affected the results of these data but may have affected the final BMI measurement of a subset of participants. Finally, the sample size and missing data limited the variables that could be included in the model.

## Conclusion

Most interventions targeting traditional energy balance related behaviors (i.e., diet and PA) have been largely ineffective to impact the obesity epidemic in children [13], suggesting a need to consider how various environments (i.e., school year and summer holiday) differentially affect children’s behavior as well as alternative determinants of change in BMI such as sleep and circadian rhythms [80]. While there is a need to replicate these findings across other climates and contexts that influence social demands such as year-long schooling [23], the results of this study indicate different behaviors may be associated with changes in children’s BMI during the school-year and summer holiday, supporting the idea that obesity prevention inventions should be tailored to in school and out of school times. During the school-year, interventions may focus on the promotion of outside time which may support exposure to light during the day as well as encourage physical activity. During extended out of school times, interventions should promote earlier bedtimes that are more consistent with sleep timing during the school-year. Finally, the CCR Model provides a testable framework for understanding the role of the school year and summer environment on children's sleep and biological rhythms and their contributions to children's weight gain. Based on the CCR Model and our current findings it may be important to look beyond the effect of PA on energy expenditure, and consider the role of sleep timing on children's accelerated summer weight gain.

## Supplementary Information

**Additional file 1.**

## Data Availability

The data are stored a Baylor College of Medicine and will be made available up request by contacting the first author.
