# Supplementary material for: Later sleep timing predicts accelerated summer weight gain among elementary school children: a prospective observational study
Source: Int J Behav Nutr Phys Act. 2021 Jul 12;18:94. doi: 10.1186/s12966-021-01165-0 (PMC8273994; doi:10.1186/s12966-021-01165-0)
Supplement: Supplementary file 1 — Additional file 1. [file 12966_2021_1165_MOESM1_ESM.docx]

**eTable 1. Children’s Height and Weight Status during the School-Year and Summer by Sex**

|  | **Baseline Fall Semester 2016**  **n=95 (Males= 51, Females=44)** | | | | | **End of Spring Semester 2017**  **n=95 (Males= 51, Females=44)** | | | | | **Beginning of Fall Semester 2017**  **n=91 (Males= 47, Females=44)** | | | |
| --- | --- | --- | --- | --- | --- | --- | --- | --- | --- | --- | --- | --- | --- | --- |
|  | Mean | SD | Min | Max | Mean | | SD | Min | Max | Mean | | SD | Min | Max |
| **Height (cm)^a^** | 121.94^■^ | 7.64 | 104.45 | 141.80 | 125.11^■^ | | 7.71 | 107.55 | 144.55 | 127.36^■^ | | 7.74 | 109.90 | 148.05 |
| Male | 121.84 | 7.58 | 104.75 | 141.20 | 125.16 | | 7.62 | 107.80 | 144.05 | 127.34 | | 7.52 | 110.20 | 145.70 |
| Female | 122.07 | 7.80 | 104.45 | 141.80 | 125.06 | | 7.89 | 107.55 | 144.55 | 127.39 | | 8.06 | 109.90 | 148.05 |
| **Weight (kg)^a^** | 25.14^▲^ | 5.69 | 16.45 | 42.30 | 26.94^▲^ | | 6.44 | 16.80 | 47.10 | 28.52^▲^ | | 7.18 | 17.40 | 51.00 |
| male | 24.88 | 5.56 | 16.60 | 42.30 | 26.64 | | 6.42 | 16.80 | 47.10 | 27.80 | | 6.84 | 17.40 | 51.00 |
| female | 25.45 | 5.88 | 16.45 | 36.90 | 27.28 | | 6.52 | 17.73 | 41.20 | 29.29 | | 7.53 | 18.30 | 44.50 |
| **BMI^a,b^** | 16.73^♦^ | 2.34 | 13.17 | 23.58 | 17.01^♦^ | | 2.63 | 13.15 | 24.85 | 17.38^♦^ | | 3.01 | 13.33 | 25.87 |
| male | 16.58 | 2.13 | 14.16 | 23.58 | 16.82 | | 2.53 | 13.15 | 24.85 | 16.95 | | 2.73 | 13.33 | 24.85 |
| female | 16.89 | 2.59 | 13.17 | 23.31 | 17.24 | | 2.74 | 13.57 | 23.86 | 17.83 | | 3.25 | 13.50 | 25.87 |
| **Standardized BMI** | 0.41 | 0.99 | -1.95 | 2.49 | 0.42 | | 1.01 | -2.45 | 2.41 | 0.44 | | 1.06 | -2.16 | 2.53 |
| male | 0.41 | 0.94 | -1.16 | 2.27 | 0.39 | | 1.01 | -2.45 | 2.30 | 0.35 | | 1.05 | -2.16 | 2.39 |
| female | 0.41 | 1.06 | -1.95 | 2.49 | 0.46 | | 1.03 | -1.49 | 2.41 | 0.55 | | 1.07 | -1.58 | 2.53 |
| **BMI Percentile** | 60.84 | 28.52 | 2.56 | 99.36 | 60.90 | | 28.28 | 0.71 | 99.20 | 60.90 | | 29.28 | 1.54 | 99.43 |
| male | 60.95 | 27.72 | 12.30 | 98.84 | 59.99 | | 27.39 | 0.71 | 98.93 | 58.37 | | 28.75 | 1.54 | 99.16 |
| female | 60.71 | 29.75 | 2.56 | 99.36 | 61.96 | | 29.56 | 6.81 | 99.20 | 63.61 | | 29.94 | 5.71 | 99.43 |

^a^Significant effect of time: Height: *F*(1.745,89) = 1584.634, *p* < 0.001; Weight: *F*(1.745,89) = 200.037, *p* < 0.001; BMI: *F*(1.42,89) = 28.405, *p* < 0.001

^b^Significant time x sex interaction: BMI: *F*(1.42,89) = 24.466, *p* < 0.05

Numbers sharing the same symbol are significantly different from each other.

**eTable 2. Children’s Sleep Schedules, Physical Activity Level, Light Exposure, and Weight Status during the School-Year and Summer by Sex**

|  | **School-year (n=95)** | | | | **Summer (n=95)** | | | |
| --- | --- | --- | --- | --- | --- | --- | --- | --- |
|  | Mean | SD^1^ | Minimum | Maximum | Mean | SD | Minimum | Maximum |
| **Sleep Midpoint^a^** | 2:17 AM | 0.65 | 12:39 AM | 4:09 AM | 3:43 AM | 1.44 | 1:29 AM | 8:19 AM |
| male (n=51) | 2:12 AM | 0.69 | 12:39 AM | 4:09 AM | 3:30 AM | 1.39 | 1:29 AM | 8:19 AM |
| female (n=44) | 2:23 AM | 0.61 | 1:00 AM | 3:45 AM | 3:59 AM | 1.47 | 1:44 AM | 7:18 AM |
| **Bedtime^a^** | 9:42 PM | 0.79 | 6:51 PM | 12:33 AM | 11:05 PM | 1.51 | 7:46 PM | 3:52 AM |
| male | 9:38 PM | 0.84 | 6:51 PM | 12:33 AM | 10:56 PM | 1.44 | 7:54 PM | 3:52 AM |
| female | 9:48 PM | 0.71 | 7:10 PM | 11:01 PM | 11:16 PM | 1.58 | 7:46 PM | 3:49 AM |
| **Wake Time^a,b^** | 6:51 AM | 0.61 | 5:51 AM | 8:36 AM | 8:10 AM | 1.40 | 5:53 AM | 12:38 PM |
| male | 6:45 AM | 0.62 | 5:45 AM | 8:37 AM | 7:52 AM | 1.32 | 5:53 AM | 12:38 PM |
| female | 6:59 AM | 0.59 | 5:59 AM | 8:35 AM | 8:31 AM | 1.43 | 6:29 AM | 11:24 AM |
| **Sleep Duration (hrs)^a^** | 7.86 | 0.50 | 6.44 | 8.77 | 7.66 | 0.67 | 5.67 | 9.50 |
| male | 7.81 | 0.53 | 6.44 | 8.66 | 7.55 | 0.64 | 5.67 | 8.94 |
| female | 7.91 | 0.46 | 7.00 | 8.77 | 7.80 | 0.69 | 6.01 | 9.50 |
| **Sedentary Behavior (hrs)^a,c^** | 7.44 | 1.16 | 4.51 | 9.96 | 7.98 | 1.67 | 2.08 | 14.02 |
| male | 7.39 | 1.13 | 4.92 | 9.82 | 8.27 | 1.88 | 2.08 | 14.02 |
| female | 7.50 | 1.19 | 4.51 | 9.96 | 7.63 | 1.32 | 4.83 | 11.33 |
| **Light PA (hrs)^a,b,c^** | 6.03 | 0.83 | 3.78 | 7.93 | 5.08 | 1.31 | 0.09 | 8.21 |
| Male | 6.04 | 0.82 | 3.78 | 7.93 | 4.72 | 1.33 | 0.09 | 6.37 |
| female | 6.03 | 0.84 | 4.04 | 7.91 | 5.49 | 1.16 | 3.01 | 8.21 |
| **Moderate to Vigorous PA (hrs)^a,b^** | 1.13 | 0.43 | 0.41 | 2.67 | 1.00 | 0.51 | 0.00 | 2.78 |
| male | 1.24 | 0.47 | 0.41 | 2.67 | 1.11 | 0.59 | 0.00 | 2.78 |
| female | 1.01 | 0.35 | 0.46 | 1.84 | 0.88 | 0.37 | 0.23 | 1.92 |

^1^ All standard deviations are shown in hours.

^a^Significant effect of Time: Sleep midpoint: *F*(1,93) = 150.7, *p* < 0.001; Bedtime: *F*(1,93) = 113.379, *p* < 0.001; Wake Time: *F*(1,93) = 129.463, *p* < 0.001; Sleep Duration: *F*(1,93) = 9.833, *p* < 0.01; Sedentary Behavior: *F*(1,93) = 9.903, *p* < 0.01; Light PA: *F*(1,93) = 57.034, *p* < 0.001; Moderate to Vigorous PA: *F*(1,93) = 6.875, *p* = 0.01

^b^Significant effect of sex: Wake Time: *F*(1,93) = 5.579, *p* < 0.05; Light PA: *F*(1,93) = 9.928, *p* < 0.01; Moderate to Vigorous PA: *F*(1,93) = 8.078, *p* < 0.01

^c^Significant time x sex interaction: Sedentary Behavior: *F*(1,93) = 5.367, *p* < 0.05; Light PA: *F*(1,93) = 9.928, *p* < 0.01

**eTable 3.** Predictors of Change in BMI during the School-Year and Summer

| Model Parameter | *Estimate* | *SE* | *DF* | *t* | *p*^a^ | *95% CI* | |
| --- | --- | --- | --- | --- | --- | --- | --- |
|  |  |  |  |  |  | *LL* | *UL* |
| Intercept | 11.26 | 3.34 | 73.10 | 3.37 | 0.00 | 4.61 | 17.91 |
| ^b^Chronotype: Definitely a Morning Type | 0.46 | 1.40 | 72.90 | 0.33 | 0.75 | -2.33 | 3.24 |
| Chronotype: Rather a Morning Type than an Evening Type | 1.05 | 1.37 | 72.80 | 0.77 | 0.45 | -1.67 | 3.77 |
| Chronotype: Neither a Morning nor an Evening Type | 0.81 | 1.48 | 72.70 | 0.55 | 0.59 | -2.14 | 3.76 |
| Chronotype: Rather an Evening Type than a Morning Type | 0.50 | 1.39 | 72.90 | 0.36 | 0.72 | -2.28 | 3.27 |
| Chronotype: Definitely an Evening Type | 1.27 | 1.85 | 73.00 | 0.68 | 0.50 | -2.42 | 4.95 |
| cRace: African American | -0.99 | 1.04 | 73.60 | -0.95 | 0.35 | -3.05 | 1.08 |
| Race: Caucasian | -0.55 | 0.93 | 73.80 | -0.60 | 0.55 | -2.40 | 1.29 |
| Race: Asian | -1.87 | 1.02 | 73.70 | -1.82 | 0.07 | -3.91 | 0.18 |
| Race: Other | 1.01 | 1.62 | 73.10 | 0.62 | 0.54 | -2.22 | 4.24 |
| Sex | -0.16 | 0.60 | 74.90 | -0.27 | 0.79 | -1.35 | 1.03 |
| Age-BL^d^ | 0.41 | 0.32 | 73.00 | 1.29 | 0.20 | -0.22 | 1.05 |
| **Age-P1^e^** | **0.04** | **0.01** | **92.70** | **4.14** | **<.0001** | **0.02** | **0.07** |
| **Age-P2^f^** | **0.05** | **0.02** | **88.60** | **2.40** | **0.02** | **0.01** | **0.10** |
| **Sex x Age-P2** | **0.06** | **0.03** | **87.20** | **2.00** | **0.05** | **0.00** | **0.13** |
| Total Sleep Time-School | -0.01 | 0.01 | 73.40 | -0.63 | 0.53 | -0.02 | 0.01 |
| Total Sleep Time-Summer | 0.01 | 0.01 | 73.20 | 1.72 | 0.09 | 0.00 | 0.03 |
| Variability of Sleep Midpoint (RMSSD^g^)-Summer | 0.00 | 0.01 | 73.00 | 0.36 | 0.72 | -0.01 | 0.02 |
| Sleep Midpoint-Summer | 0.01 | 0.00 | 75.00 | 1.50 | 0.14 | 0.00 | 0.01 |
| **Age-P2 x Sleep Midpoint-Summer** | **0.00** | **0.00** | **88.40** | **2.16** | **0.03** | **0.00** | **0.00** |
| Percent Time Spent in Sedentary Behavior-School | -0.01 | 0.05 | 73.20 | -0.12 | 0.90 | -0.10 | 0.09 |
| Percent Time Spent in Moderate to Vigorous PA^h^-School | 0.10 | 0.13 | 73.70 | 0.79 | 0.43 | -0.16 | 0.36 |
| Model Parameter | *Estimate* | *SE* | *DF* | *t* | *p*^a^ | *95% CI* | |
|  |  |  |  |  |  | *LL* | *UL* |
| Percent Time Spent in Sedentary Behavior-Summer | -0.01 | 0.04 | 72.80 | -0.19 | 0.85 | -0.08 | 0.07 |
| Percent Time Spent in Moderate to Vigorous PA-Summer | -0.16 | 0.11 | 72.90 | -1.40 | 0.17 | -0.08 | 0.07 |
| Percentage of Time Outdoors-School | -0.07 | 0.14 | 75.60 | -0.51 | 0.61 | -0.34 | 0.20 |
| Percentage of Time Outdoors-Summer | 0.05 | 0.11 | 73.10 | 0.45 | 0.65 | -0.17 | 0.27 |
| **Age-P1 x Percentage of Time Outdoors-School** | **-0.01** | **0.01** | **91.90** | **-2.48** | **0.01** | **-0.02** | **0.00** |

These are the results from a single growth curve model used to estimate individual differences in intercepts and slopes for BMI during the school-year and the summer. This model explained 25.47% of BMI variation across the study period (R^2^ = .2547).

^a^Significance level was set at *p*< .05. ^b^Reference category: I don’t know; ^c^Reference Category: Mixed Race; ^d^BL: Baseline Fall 2016; ^e^P1: End of the School-Year 2017; ^f^P2: Beginning of the School-Year 2017; ^g^RMSSD: Root Mean Square of Successive Differences; ^h^PA: Physical Activity

**eFigure 1. Study Flow Diagram**
